# A consensus statement for trauma surgery capacity building in Latin America

**DOI:** 10.1186/s13017-021-00347-2

**Published:** 2021-01-30

**Authors:** Mohini Dasari, Erica D. Johnson, Jorge H. Montenegro, Dylan P. Griswold, Maria Fernanda Jiménez, Juan Carlos Puyana, Andres M. Rubiano, On behalf of the Cartagena Consensus, Juan C. Puyana, Andres M. Rubiano, Jorge H. Montenegro, Mohini Dasani, María V. Rodríguez, Sabrina Asturias, Gustavo Miguel Machain, Ezequiel Monteverde, Paulo Roberto Lima Carreiro, Raul Augusto Echeverri, Edgar B. Rodas, Lina V. Mata, Carlos A. Ordoñez, José Miguel Salmerón, Gustavo Salas, Rodolfo Farfán Jaime, Cristina Rodríguez, Amaury García, Melissa Saul, Marcos D. Pereira Dohmen, Emily Reid Rodríguez, José Luis Coronado, Kee Park, Teri Reynolds, Walter Johnson

**Affiliations:** 1 University of Pittsburgh School of Medicine, Pittsburgh, PA USA; 2 MEDITECH Foundation, Santiago de Cali, Valle de Cauca, Colombia; 3 NIHR Global Health Research Group on Neurotrauma, University of Cambridge, Cambridge, UK; 4 Division of Neurosurgery, Department of Clinical Neurosciences, Addenbrooke’s Hospital & University of Cambridge, Cambridge, UK; 5 Stanford School of Medicine, Stanford, CA USA; 6 Department of Surgery, Hospital Universitario Méderi, Bogota, Colombia; 7 Department of Surgery, University of Pittsburgh Medical Center, Pittsburgh, PA 15213 USA; 8 Institute of Neuroscience, Universidad El Bosque, Bogotá, Colombia

**Keywords:** Trauma, Trauma registry, Injury surveillance, Latin America, Consensus statements, Acute care, TBI, LMICs

## Abstract

**Background:**

Trauma is a significant public health problem in Latin America (LA), contributing to substantial death and disability in the region. Several LA countries have implemented trauma registries and injury surveillance systems. However, the region lacks an integrated trauma system. The consensus conference’s goal was to integrate existing LA trauma data collection efforts into a regional trauma program and encourage the use of the data to inform health policy.

**Methods:**

We created a consensus group of 25 experts in trauma and emergency care with previous data collection and injury surveillance experience in the LA. region. Experts participated in a consensus conference to discuss the state of trauma data collection in LA. We utilized the Delphi method to build consensus around strategic steps for trauma data management in the region. Consensus was defined as the agreement of ≥ 70% among the expert panel.

**Results:**

The consensus conference determined that action was necessary from academic bodies, scientific societies, and ministries of health to encourage a culture of collection and use of health data in trauma. The panel developed a set of recommendations for these groups to encourage the development and use of robust trauma information systems in LA. Consensus was achieved in one Delphi round.

**Conclusions:**

The expert group successfully reached a consensus on recommendations to key stakeholders in trauma information systems in LA. These recommendations may be used to encourage capacity building in trauma research and trauma health policy in the region.

## Background

Trauma is a significant global health challenge. Each year, more than 5 million die because of injury. Around 1.2 million deaths worldwide are due to motor vehicle collisions, and road traffic collisions are a leading cause of death and disability among the young in low- and middle-income countries (LMICs). Injuries result in an additional 182 million disability-adjusted-life-years lost and 500 years of annual productivity lost per 100,000 inhabitants worldwide [1]. The injury burden is high LMICs, where an estimated 90% of injury-related deaths occur [2].

Moreover, social and economic costs are the highest for these developing societies [3]. In particular, Latin America, Central Africa, and South Asia have a remarkably high death and disability rates due to social violence and road traffic incidents (RTIs) [4, 5]. However, the actual social and economic cost of trauma is often difficult to quantify due to the lack of robust epidemiological data in many world regions [5, 6].

The World Health Organization (WHO) Department of Violence, Injury Prevention and Disability promotes actions to improve injured patients’ healthcare worldwide. A significant focus of these efforts has emphasized regional and international data collection for research and quality improvement. Implementing trauma registries and developing quality improvement programs is strongly recommended, especially in LMIC’s [7]. The development of these initiatives is essential to create strategies to improve trauma care systems. Information about the local “ecology of care” elucidates risk factors, injury characteristics, healthcare organization, and other social and political factors impact injury outcomes. This preliminary information helps to define the burden of trauma for a given region of the world. In time, these data may inform the development of public health policies specifically tailored to improve trauma care systems in less developed regions [8]. In recent years, many Latin American countries have made efforts to design, implement, and evaluate trauma data collection and quality improvement programs [9–11].

There are currently several trauma registry projects in Latin America, ranging from epidemiological injury surveillance systems to more formal trauma registries. However, these projects exist in isolation and are categorized as institutional, multi-institutional, or national. The Latin American region lacks a cohesive trauma network with a common objective. Integrating these efforts into a regionally inclusive endeavor is considered a pivotal step to develop capacity in trauma and acute care research, especially in partnership with global health initiatives from high-income countries (HICs) [12, 13].

The Global Health Office of the Department of Surgery at the University of Pittsburgh Medical Center and the Trauma & Emergency Care research group of MEDITECH Foundation, a collaborative research center in Colombia, organized a consensus conference of individuals and institutions engaged in trauma and emergency care initiatives across Latin America to promote capacity building for trauma research and quality improvement initiatives in the region. The meeting was sponsored by a grant from the Fogarty International Center of the United States National Institutes of Health (NIH). The conference’s goal was to develop a joint roadmap for trauma and emergency care in Latin America, promote collaboration and information sharing between the many existing data collection efforts currently carried out in the region, and encourage quality improvement and healthcare policy change.

## Materials and methods

### The consensus conference

The consensus conference took place in the city of Cartagena de Indias, Colombia, in February of 2017. Over 25 regional experts attended this meeting, each with experience in individual and institutional trauma and acute care data collection efforts. Experts represented trauma information system efforts in Guatemala, Nicaragua, Paraguay, Honduras, Brazil, Colombia, Bolivia, Ecuador, Argentina, Chile, the Dominican Republic, and El Salvador. Through a review of authors published in peer-reviewed journals and a review of positional leaders and other contacts with expertise in the field, the panel was selected. Representatives from the USA trauma centers working in Latin America, the World Health Organization, and the Global Surgery and Social Change Program of Harvard University also participated in the conference. The conference’s research endeavors were managed jointly by a methodological group consisting of the qualitative research team from the Foundation for Medical and Technical Education and Research in Emergency Care (MEDITECH) and qualitative investigators from the University of Pittsburgh.

### Plenary sessions

The consensus conference commenced with a discussion of trauma registries and injury surveillance systems and their role in developing trauma care capacity worldwide. A plenary session presented the experts’ individual experiences with trauma registries and injury surveillance registries on the first day. Nine initiatives were presented, including projects from Guatemala, Honduras, El Salvador, Paraguay, Ecuador, Colombia, and Argentina. On the second day, international experts from the University of Pittsburgh, Harvard University, and the World Health Organization gave lectures on registries’ role in global neurosurgery.

Following the plenary discussion, regional experts were divided into two working groups for panel discussions. The panel discussions were focused on future action steps based on the results of existing experiences with the heterogeneous trauma registries and injury surveillance systems. Panel discussions began with reviewing the available scientific evidence, followed by a discussion of local needs, to contextualize these aspects into a regional reality. Each working group was assigned a specific aim (Table 1).

**Table 1:** Cartagena Consensus Conference Working Group specific aims and assigned tasks

Working group 1	Developing leadership to improve the collection and availability of health data
o Task1	What do you think is the best process to improve the collection and availability of health data on trauma in your region? (describe the step by step)
o Task 2	What do you think is the best process to induce a change in the culture of health information use for political decision making regarding the organization of trauma care systems? (describe the step by step)
Working group 2	Harmonizing existing data collection efforts and supporting international trauma research collaborations
o Task 1	What do you think is the best process to harmonize existing data collection efforts, with the goal of performing joint data analysis? (describe the step by step)
o Task 2	How can we best create network collaborations and coordinate support for international research in trauma? (describe the step by step)

#### Group 1 (government and health policy)

To provide recommendations for ways to develop leadership to improve the collection and availability of health data and create a culture of use of these data for health policy decision-making.

#### Group 2 (academia)

To provide recommendations to leverage existing trauma registries to optimize joint data analysis, encourage collaboration, and coordinate international research opportunities in trauma.

### The Delphi technique

The research team employed the Delphi method to build a consensus statement on research, quality improvement, and trauma initiatives in Latin America based on discussion from the working groups. The Delphi technique is an iterative, sequential process to create consensus on a topic through structured discussion or survey [14]. This technique has previously been used to develop guidelines and achieve consensus in trauma care [15, 16]. The Delphi technique encourages structured group communication, allowing the expert panel to apply their expertise to a complex problem.

In preparation for the meeting, information on the Delphi methodology and the planned tasks was delivered to the experts before the plenary sessions. The experts reviewed this material to prepare for table discussion of their assigned tasks. After plenary sessions on the first day, experts split into working groups to discuss the tasks, intending to develop a series of future actions to accomplish their tasks. Following plenary sessions on the second day of the conference, the groups met to reach a consensus on an approach to data collection in trauma and emergency surgery in Latin America. Each working group presented their proposed stepwise approach to each task. The unification of every task’s approach was built by a consensus of at least 70% agreement between all the participants. The methodological group facilitated the experts’ discussions in two task-force subgroups and during the conference consensus process. The participants took responsibility for proposing the best options based on their knowledge, previous review of the related documents, and their own real field experience at the country level. This research aimed to compile a description of current trauma data collection efforts in the region and develop a consensus statement comprised of recommendations to address the two groups’ working aims.

The consensus statement’s ultimate aim was to improve patient safety and enhance trauma quality of care while simultaneously supporting the establishment of organized trauma systems in the region. At the end of the conference, the group compiled the concerted response results in a summary entitled “The Cartagena Declaration for Trauma and Injuries Data Collection in Latin America.” The final product of this consensus exercise endorsed several actionable items to improve the data collection process and promote data collection for public policy development.

## Results and discussion

Regional experts presented their experiences with trauma data collection experiences in Latin America, including barriers and facilitators to implementation. The working groups spent 1 day in table discussion, developing a list of steps to achieve their stated tasks. Each group achieved at least 70% agreement on the series of steps necessary to achieve their stated goals.

On the second day of the congress, representatives from each group presented their proposed future action plans to the full congress. During the group discussion, the experts agreed that the best way to accomplish their stated tasks would be to engage ministries of health, scientific societies, academic institutions, and other groups whose actions could impact the collection and use of health data in trauma. Therefore, the consensus group used the steps outlined in their small group discussion to develop the *Cartagena Declaration for Trauma and Injuries Data Collection in Latin America*, a series of recommendations to Ministries of Health, academic bodies, and scientific societies to improve the state of trauma data collection and to create a culture of use of the data in health policy in Latin America.

### A summary of existing trauma data collection efforts in Latin America

Experts from participating countries presented their experiences with regional data collection, describing their strengths and weaknesses, and discussed barriers and pitfalls to their implementation.

#### Guatemala: the electronic health record project

This project started in Guatemala City in 2014 with the implementation of an electronic data collection tool in a public hospital. The project was part of a research grant sponsored by the NIH Fogarty Institute and supported by the Global Health Office of the Department of Surgery at the University of Pittsburgh. Variables collected through this system included essential demographic elements at admission, operative notes, and necessary outcome measurements. Experiences with the EHR project illustrated several barriers in the process of implementing a data collection tool. The main barrier identified was related to the transition of information from paper to an electronic record. Guatemalan investigators considered that medical errors could increase the cost of care and analysis of appropriate medical data can improve the patient’s care [17].

#### Paraguay: the electronic health record project

This project began in Asunción City in 2014 with the implementation of an electronic data collection tool in an acute care surgery service at one public hospital. The project was also part of a research grant sponsored by the NIH Fogarty Institute and supported by the Global Health Office at the Surgery Department at the University of Pittsburgh. Variables collected through this system included essential demographic elements at admission, operative notes, and necessary outcome measurements. The main barriers to implementing this project related to the lack of hospital WiFi connectivity, required for the operation of the electronic data collection tool. Additionally, equipment safety and theft presented barriers to EHR implementation [17, 18].

#### Honduras: the external injury surveillance system

This project was implemented as a standardized electronic injury surveillance system in Hospital Escuela Universitario of Honduras in Tegucigalpa over 10 years. This initiative resulted from a consortium collaboration that included Harvard University and the International Committee of the Red Cross. The main obstacles encountered during the implementation of this system included the absence of a local organized trauma system, which affected the type of variables that could be collected. Injury surveillance endeavors are not trauma registries; therefore, the data collected on the epidemiology of injury and violence lacks any clinical outcomes that would be otherwise pivotal for assessing patients’ quality of care and stratification according to injury severity scores. Other issues encountered included an absence of government support and difficulties integrating public health surveillance teams with local clinicians and surgeons to facilitate patient care [19, 20].

#### Brazil: the trauma registry project of hospital Joao XXIII

This project was developed in the major trauma center in Belo Horizonte City in Minas Gerais state. It was built to fill information gaps in DATASUS, the Brazilian national health data information system. This national health data surveillance system correlates trauma deaths with basic demographic data. The trauma group of Hospital Joao XXIII started a local trauma registry to supplement DATASUS by collecting medical and surgical data associated with trauma patients’ outcomes. They found that the quality of data in local medical records was low, especially for prehospital care and emergency room data. Currently, the registry is non-functional due to the absence of a dedicated budget for technical support and personnel for data registry operation [21, 22].

#### Colombia, Bolivia, and Ecuador: the pan-American trauma registry

This program, supported by the Pan American Trauma Society and the International Trauma Development Systems program of Virginia Commonwealth University, started data collection in three different countries, supported by local hospital initiatives in Santa Cruz de la Sierra (Bolivia), Cuenca (Ecuador), and in Cali and Medellin (Colombia). The most extensive trauma data set so far has been collected in Cali. This research data collection has allowed the Cali research group to generate significant contributions to the trauma literature in Latin America. The Cali group’s experience is now consistently shared worldwide, contributing to the medical literature in diverse subjects, including complex abdominal and thoracic trauma. Barriers to the Pan-American Trauma Registry include lack of funding and long-term sustainability of the project. Some centers are reliant on local grants to support the registry’s continuity [23–25].

#### Argentina: the trauma registry project

This project was developed by Foundation Trauma, a private foundation in Buenos Aires City. This privately funded initiative began in 2009 as part of an inclusive program uniting 14 hospitals of Buenos Aires province. The registry includes robust trauma data variables, including trauma scores (AIS, GOS, RTS, ISS, NISS, TRISS) for quality and mortality analysis. In addition to data collection, the foundation supports educational programs targeting trauma management and trauma quality improvement across the network. Difficulties with implementation stem from variability in the implementation process among hospitals. For example, some institutions use electronic medical records, while others rely upon paper-based clinical records. However, the amount of surveillance and clinical care data collected through this program has allowed the group to generate critical information for public policy development at the local level. In addition, they have implemented trauma quality improvement programs supported by WHO initiatives [26].

#### El Salvador: SILEX

The online surveillance program for external injuries. Sistema de Información de Lesiones de Causa Externa (SILEX) is a government initiative supported by the Pan American Health Organization and the United States Centers for Disease Control (CDC). Established in 2002 as a trauma surveillance program, SILEX continues to collect data in several hospitals in El Salvador through a web platform. The program generates graphic reports and tables of descriptive statistics for the Ministry of Health. However, the absence of an organized, regionalized trauma system of care impedes a connection with a trauma registry that could generate public policies after outcome evaluations [27]. In addition, the main difficulty faced by SILEX has been the maintenance of data quality and an inability to perform in-depth clinical analysis of health data due to the heavy workload of the epidemiological data team.

#### Colombia: the LATINO project

This multicentric neurotrauma registry initiative called the LATINO project is a regional registry focused in neurotrauma. The program started as part of the data collection registry for an R21 grant supported by the Fogarty International Institute of the NIH aimed at capacity building in decompressive craniectomy research in Colombia. The registry is hosted through a local server at Universidad El Bosque in Bogotá and is supported by the research team of MEDITECH Foundation. This registry aims to understand the ecology of neurotrauma care in Latin America. The registry includes demographic, emergency, surgical, and critical care data associated with outcomes after traumatic brain and spinal cord injuries. Pilot testing of the registry, involving data collection from 150 neurotrauma patients at two centers in Colombia, is now complete. The registry’s goal is to expand data collection to at least additional countries in Latin America to create a multi-national patient data registry for research and quality improvement [28, 29].

#### Ecuador: trauma information system

The city of Guayaquil’s experience was presented, including a limited experience with electronic medical records usability in some health centers of the city, and new projects for emergency dispatch centers supported by the government locally. Trauma education programs, including prehospital care teams, were critical aspects of the data collection process in Ecuador.

#### The United States of America: the committee on trauma

The United States of America: The Committee on Trauma (COT) of the American College of Surgeons (ACS) presented a chapter dedicated to data collection on trauma care in the “Resources for Essential Trauma Care” book, promoting this chapter as an instrument to build capacity for trauma systems development [30]. This document is now being translated into Spanish. The University of Pittsburgh also presented their experience with the Pennsylvania Trauma Registry as an example of a robust trauma quality data collection enterprise in a high-income setting [31]. They also provided a lecture on the Common Data Elements project from the United States National Institutes of Health [32], describing the advantages of this approach for further international multicentric clinical research initiatives.

#### The World Health Organization and the global surgery for social change program

The World Health Organization (WHO) and the Global Surgery for Social Change Program at Harvard University gave three online lectures describing several global health alliances centered around data collection initiatives in global surgical care and trauma and emergency care, including examples of partnerships between international medical societies to increase the quality of data collection worldwide [33]. The WHO presented its global burn registry initiative and described the minimal data set initiative for globalized trauma registries [34].

### The consensus statement

The working group on injury surveillance and trauma registry initiatives in the Latin American Region unanimously decided to create a consensus statement to reflect their discussion results. Each item on the statement met the consensus definition, defined before starting the conference as ≥ 70% agreement.

## The Cartagena declaration for trauma and injuries data collection in Latin America

Given that Latin America is a region severely affected by both intentional injuries (violence) and unintentional injuries (traffic accidents, falls, burns, drowning), and, also considering that most countries in this region lack trauma registries which prevent them from quantifying and addressing the problem, we affirm the following:

●Trauma is a public health problem in Latin America.

●Trauma has substantial social and economic impact due to its high mortality rate and associated physical and mental disability.

●Trauma burden represents a significant percentage of national healthcare spending in the region.

●Medical and surgical care is an integral part of public health care.

●Trauma is a disease whose impact can be reduced through government and community programs focused on prevention.

●Mortality and disability can be prevented by implementing national trauma systems where the trauma registry acts as a fundamental data collection tool.

●Health decisions have a real impact when they are based on reliable and complete population statistics.

●Decision-making on quality improvement in trauma care must be based on the available, local statistical data.

●Consequently, Latin American health professionals, including physicians, surgeons, intensivists, pediatricians, psychiatrists, epidemiologists, public health specialists, computer science specialists, mathematicians, and statisticians, gathered in the city of Cartagena de Indias in Colombia have arrived at a consensus.

We declare the following:

●To the ministries of health

Ministries of Health of Latin America should be made aware of the need to implement national trauma systems, including reliable, precise, and sustainable trauma registries that monitor trauma care processes.

Health policies should be generated from local statistics derived from reliable records and based on strong scientific evidence.

Trauma registries must comply with international standards and include quality indicators that identify improvement opportunities and share best practices.

These registries should generate statistical analysis to inform decision-making on trauma management to improve trauma patient care quality and safety.

That trauma registries should comply with international standards for patient data registries and common data elements and should include quality indicators that identify quality improvement and benchmarking opportunities.

These registries should become sources of data for statistical analyses to inform decisions that improve the quality and safety of patients with a traumatic injury.

These registries must become part of the National Trauma Care System in countries across Latin America.

●To the academic bodies

A culture of health data collection, medical record keeping, and statistical analysis should be an integral part of the curriculum for health professionals, beginning with the earliest years of training.

Academic bodies should promote scientific investigation to generate knowledge that can be used to improve the injured patient’s care.

Academic bodies should support Ministries of Health and other health institutions in the maintenance of trauma registries and the identification of variables and quality indicators that are necessary, sensitive, and specific.

●To scientific societies

Scientific societies should promote awareness of the implementation and maintenance of the trauma registries among Governments and Ministries of Health.

Scientific societies must ensure that the academy fulfills its obligations regarding trauma care systems and trauma registries through health professionals’ education.

Scientific societies must engage in the development of standardization and accreditation processes for health facilities across varying levels of complexity that are engaged in the trauma patient’s care.

Scientific societies must disseminate official institutional standards for the patient’s medical and surgical care with a traumatic injury.

## Conclusions

Injury surveillance and trauma registry information obtained from interoperable, reliable, precise, and sustainable databases are useful sources of information for developing public policy initiatives to improve trauma patients’ care. Existing trauma registry and injury surveillance systems in Latin America highlight barriers to creating and maintaining data collection systems in the region. It is essential to combine efforts to develop long-term policies to sustain and improve national and international trauma data collection systems. A formal call is made to each country in Latin America, identifying fundamental actions by Ministries of Health, academic bodies, and scientific societies to promote health data collection and use. By harnessing existing knowledge and experiences, we may create robust partnerships to generate the change that is so urgently needed in our region, across countries so gravely affected by this epidemic.

## Data Availability

Data is presented in the manuscript. Any additional information can be requested using the corresponding author’s contact information.

## Abbreviations

LA: Latin America
LMICs: Low-and middle-income countries
HICs: High-income countries
RTIs: Road traffic incidents
WHO: World Health Organization
NIH: National Institute of Health
MEDITECH: Medical and Technical Education and Research in Emergency Care
EHR: Electronic health record
COT: Committee on trauma
ACS: American College of Surgeons
SILEX: Sistema de Información de Lesiones de Causa Externa
